# Facile synthesis and photocatalytic activity of bi-phase dispersible Cu-ZnO hybrid nanoparticles

**DOI:** 10.1186/s11671-015-0895-2

**Published:** 2015-04-23

**Authors:** Xiao Liu, HongLing Liu, WenXing Zhang, XueMei Li, Ning Fang, XianHong Wang, JunHua Wu

**Affiliations:** Key Lab of Polyoxometalate Chemistry of Henan Province, Institute of Molecular and Crystal Engineering, School of Chemistry and Chemical Engineering, Henan University, Kaifeng, 475001 China; Shangqiu Normal University, Shangqiu, HeNan Province 476000 China; Department of Materials Science and Engineering, South University of Science and Technology of China, Shenzhen, 518055 China; Pioneer Research Center for Biomedical Nanocrystals, Korea University, Seoul, 136-713 South Korea

**Keywords:** Nanoemulsion, Bi-phase dispersible, Cu-ZnO nanoparticles, Photocatalytic performance

## Abstract

Bi-phase dispersible Cu-ZnO hybrid nanoparticles were synthesized by one-pot non-aqueous nanoemulsion with the use of poly(ethylene glycol)-block-poly(propylene glycol)-block-poly(ethylene glycol) (PEO-PPO-PEO) as the surfactant. The transmission electron microscopy (TEM) and X-ray diffraction (XRD) show high crystallinity of the Cu-ZnO hybrid nanoparticles and an average particle size of ~19.4 nm. The ultraviolet–visible light absorbance spectrometry (UV–vis) and photoluminescence spectrophotometry (PL) demonstrate well dispersibility and excellent optical performance of Cu-ZnO hybrid nanoparticles both in organic and aqueous solvent. The X-ray photoelectron spectroscopy (XPS) confirms Cu^1+^ and Cu^2+^ in ZnO. The observation using Sudan red (III) as probe molecule reveals that the Cu-ZnO hybrid nanoparticles possess enhanced photocatalytic activity and stability which are promising for potential applications in photocatalysis.

## Background

Multi-constituent nanomaterials with different compositions and tailorable morphology display multiple functionalities and novel properties, showing prospective potentials in drug delivery, biological detection and sensing, imaging, separation, strong catalysis, magnetic data storage, chemotherapy agent, and many other areas [1-7]. Syntheses of such nanoparticles and investigating their various properties are hence of general interest. ZnO is a widely studied *n*-type semiconductor, with direct band gap (3.37 eV) and large exciton binding energy of 60 meV, and has promising applications in catalysis, solar cells, gas sensor, and miniaturized sensor [8-11]. However, its applications are somewhat restricted [12]. Doping ZnO with transition metal elements has been confirmed as an effective method to improve its functionality including electrical and optical properties. Among the transition metal elements, Cu is the best choice as impurity for realization of *p*-type ZnO due to the minimum size mismatch between Zn and Cu which leads to the lowest formation energy [13,14]. After nano-engineering Zn and Cu into a single entity, the nanostructure would not only possess the unique properties of the copper and the semiconductor but also generate collective new property based on the interaction between Cu and ZnO. The photocatalytic performance, magnetic, electrical, and gas-sensing properties of Cu-ZnO have been studied for their potential applications in photocatalysis, spintronics, and gas sensor [15-17]. Up to now, Cu-ZnO has been synthesized by a variety of methods such as electrochemical synthesis, co-precipitation, vapor phase transport method, and hydrothermal method [18-21].

In our research, excellent nanoparticles could be synthesized via one-pot non-aqueous nanoemulsion process aided by poly(ethylene glycol)-block-poly(propylene glycol)-block-poly(ethylene glycol) (PEO-PPO-PEO). The triblock copolymer PEO-PPO-PEO possesses many distinctive merits, such as non-charging, aqueous solubility, non-toxicity, and biocompatibility, and is widely used in various fields [22-26]. In nanoemulsion process, the PEO-PPO-PEO molecules predominantly participate in the reaction as a surfactant, even playing a role in stabilizing the nanoparticles formed and acting as the role of a reducing agent. We have previously generated long-term stable, monosized, highly crystalline Fe_3_O_4_-ZnO, Au-ZnO, Ag-ZnO, and hybrid-phase iron oxide nanoparticles [27-30]. In this paper, we report the preparation of polymer-capped Cu-ZnO hybrid nanoparticles using non-toxicity and biocompatible triblock copolymer PEO-PPO-PEO as the surfactant. The characterization demonstrates that the nanoparticles are monosized and of high crystallinity, showing excellent dispersibility and optical performance both in organic and aqueous medium. The photocatalytic behavior of the nanoparticles is evaluated using Sudan red (III) as a probe molecule. The results reveal that the nanostructured Cu-ZnO moieties unveil enhanced photocatalytic performance and stability. Therefore, the as-synthesized Cu-ZnO hybrid nanoparticles could be acted as a promising photocatalyst candidate in the degradation of organic pollutants.

## Methods

Cu-ZnO hybrid nanoparticles were prepared by one-pot non-aqueous nanoemulsion method. A typical synthesis was carried out in a 100-ml flask; 0.15 mmol (0.0393 g) of copper acetylacetonate, 1.35 mmol (0.3559 g) of zinc acetylacetonate, 0.1358 mmol (0.7878 g) of PEO-PPO-PEO, and 1.877 mmol (0.4851 g) of 1,2-hexadecanediol were mingled in 10 ml octyl ether under vigorous stirring. Firstly, the reaction mixture was heated to 125°C with 1 h and maintained for 1 h at 125°C, then rapidly heated to 280°C within 15 min and refluxed at the temperature for 1 h to complete the reaction. After cooling down to room temperature, the precipitated product was separated from the supernatant by centrifugation, which was washed with ethanol/hexane (2:1) several times, and re-dispersed in hexane for further use. For comparison, ZnO nanoparticles were prepared similarly using only zinc acetylacetonate as the precursor.

The morphology and structure of the Cu-ZnO hybrid nanoparticles were characterized by transmission electron microscopy (TEM, JEM-100II, JEOL Ltd., Tokyo, Japan) and X-ray diffraction (Philips X’Pert Pro, Philips, Amsterdam, Netherlands; *λ* = 1.54056 Å) using Cu *K*_*a*_ radiation. X-ray photoelectron spectroscopy (XPS) was carried out on a Thermo ESCALAB 250XI photoelectron spectrometer with Al *Kα* X-ray as the excitation source. The optical properties of nanoparticles were characterized by a UV-visible spectrophotometer (UV–vis near IR spectrophotometer, Hitachi U4100; Hitachi, Shanghai, China) and a photoluminescence (PL) spectrophotometer (Hitachi F7000, Japan). The FT-IR spectra were recorded at the wavenumber range of 400 to 4,000 cm^−1^ using an Avatar 360 FT-IR spectrometer (Nicolet Company, Madison, WI, USA).

The photocatalytic activity was investigated under a variety of conditions via measuring the degradation rate of a Sudan red (III) dye solution at room temperature. Sudan red (III) was prepared with a concentration of 10 mg L^−1^, by dissolving the dye powder in ethanol. The photocatalytic reaction was carried out at room temperature under UV and sunlight irradiation. A UV light 36 W UV-A tube mainly emitting at 365 nm (Philips) was used. The distance between the lamp and reaction beaker is 10 cm. The reaction was conducted with 5 mg of a catalyst dispersed in 30 mL of 10 ppm Sudan red (III) ethanol solution. Prior to irradiation, the solution was stirred in the dark for 20 min to ensure the establishment of adsorption-desorption equilibrium. After illumination, the samples (volume of each is about 3.7 mL) were withdrawn from the reaction beaker every 10 min, centrifuged at 4,800 rpm for 5 min, and filtered to remove the particles. The filtrate was then analyzed using a UV–vis spectrophotometer (Beijingpuxitongyong TU-1900, Beijing Puxi Tongyong Instrument Company, Beijing, China) to measure the absorption of Sudan red (III) at the range of 200 nm to 800 nm.

## Results and Discussion

**Figure 1:**
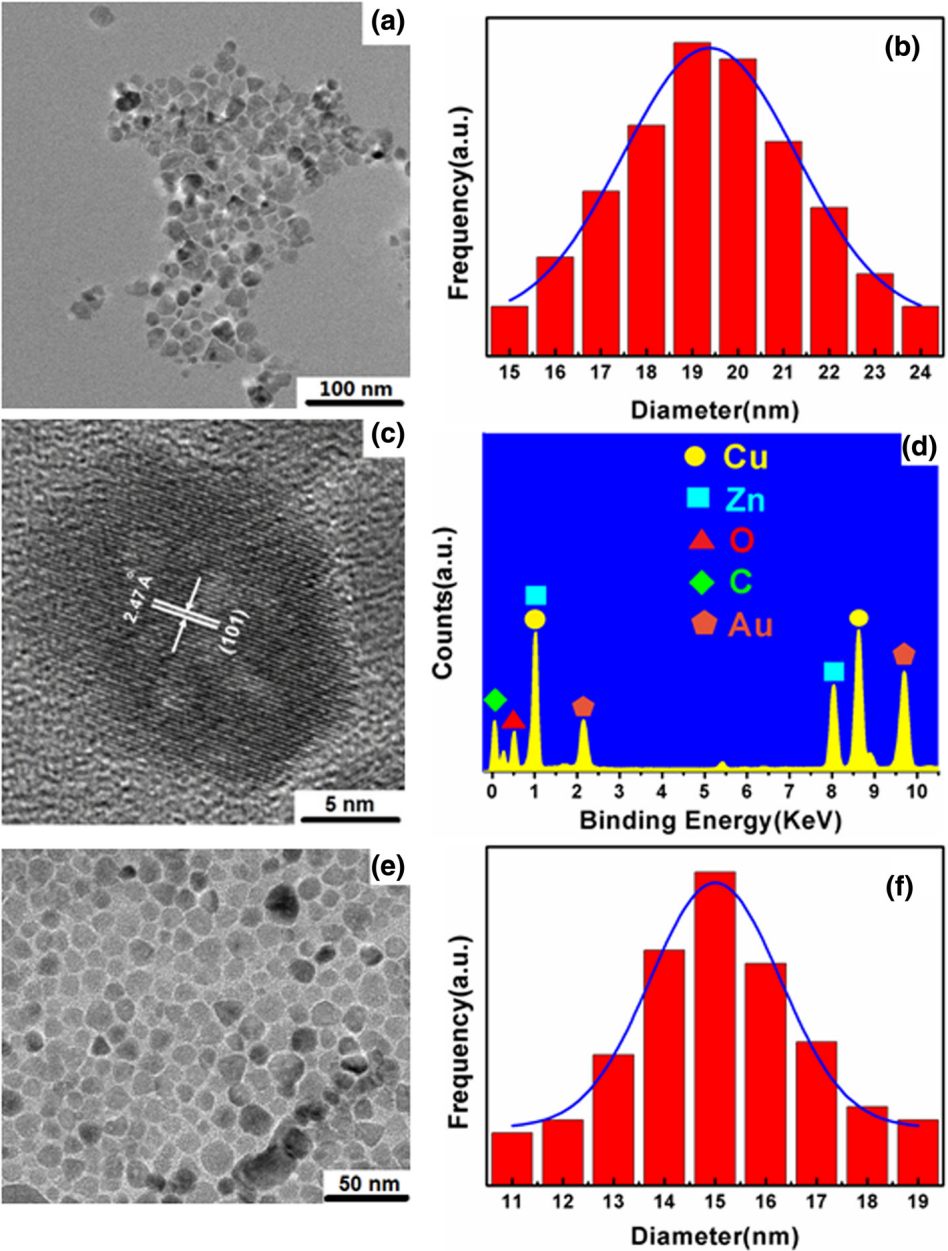
TEM analyses of the nanoparticles. **(a)** Bright-field image of Cu-ZnO nanoparticles, **(b)** particle size distribution (in histogram) with Gaussian function fit (in curve) of Cu-ZnO nanoparticles, **(c)** HRTEM of an individual Cu-ZnO nanoparticle, **(d)** point-detection EDX analysis of Cu-ZnO nanoparticles, **(e)** bright-field image of ZnO nanoparticles, and **(f)** particle size histogram with Gaussian fit of ZnO nanoparticles.

Figure 1a,e shows the morphology and particle sizes of prepared Cu-ZnO and ZnO nanoparticles recorded by TEM. Obviously, both Cu-ZnO and ZnO nanoparticles are virtually uniform and nearly spherical in shape with seldom aggregation. The histograms in Figure 1b,f reveals the size distribution of Cu-ZnO and ZnO nanoparticles, which are reasonably described by the Gaussian function, showing tight size distribution with average sizes of approximately 19.4 and 15.0 nm in diameter and standard deviation of 1.9 and 1.3 nm for Cu-ZnO and ZnO nanoparticles, respectively. The HRTEM image of a single Cu-ZnO hybrid nanoparticle is shown in Figure 1c. As labeled, the spacing of 2.47 Å indicates the projection of the ZnO (101) plane. All the lattices can be assigned to ZnO, and there is no other lattice for Cu because of the incorporation of Cu ion into the Zn lattice site [31]. Figure 1d shows a typical TEM-EDX point-detection instance for the composition, clearly showing the simultaneous presence of Zn, O, and Cu elements.

**Figure 2:**
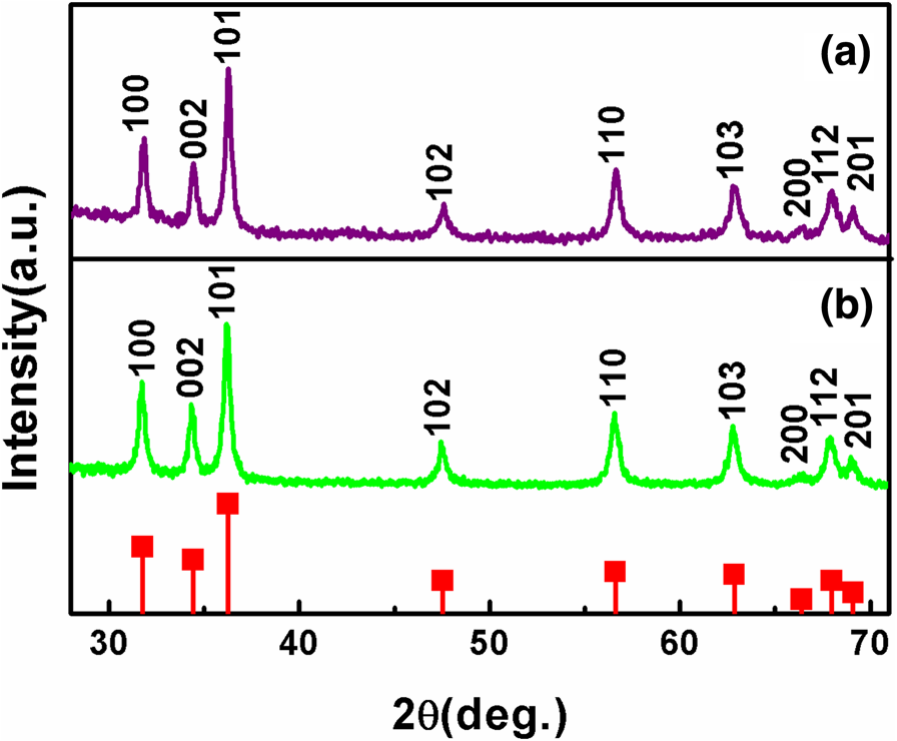
X-ray diffraction patterns of the **(a)** Cu-ZnO hybrid nanoparticles and **(b)** ZnO nanoparticles. Bar diagram for the JCPDS of ZnO (in square).

The formation of the Cu-ZnO hybrid nanoparticles was further validated by the X-ray crystal structural analysis in comparing with ZnO nanoparticles. As shown in Figure 2a, all the observed diffraction peaks of samples can be indexed to the hexagonal wurtzite structure of ZnO (JCPDS card no. 36–1451). The diffractions peaking at 31.80°, 34.40°, 36.28°, 47.58°, 56.62°, 62.92°, 66.36°, 67.96°, and 69.11° are appropriately indexed to the Cu-ZnO hybrid nanoparticles at the positions of (100), (002), (101), (102), (110), (103), (200), (112), and (201) planes. These peaks generate a slightly shift (not more than 0.1°) relative to those of ZnO nanoparticles (Figure 2b), and no other peaks corresponding to Cu related secondary or impurity phase are found, which may be due to the incorporation of Cu ion into the Zn lattice site rather than interstitial ones [32]. In addition, the average size of the Cu-ZnO hybrid nanoparticles is estimated to be approximately 18.8 nm by the Scherrer equation, supposing that the broadening of the peaks in the X-ray diffraction (XRD) pattern is predominantly due to the finite size of the nanoparticles [33]. The assessment is reasonably consistent with the statistical counting of the TEM analysis above.

**Figure 3:**
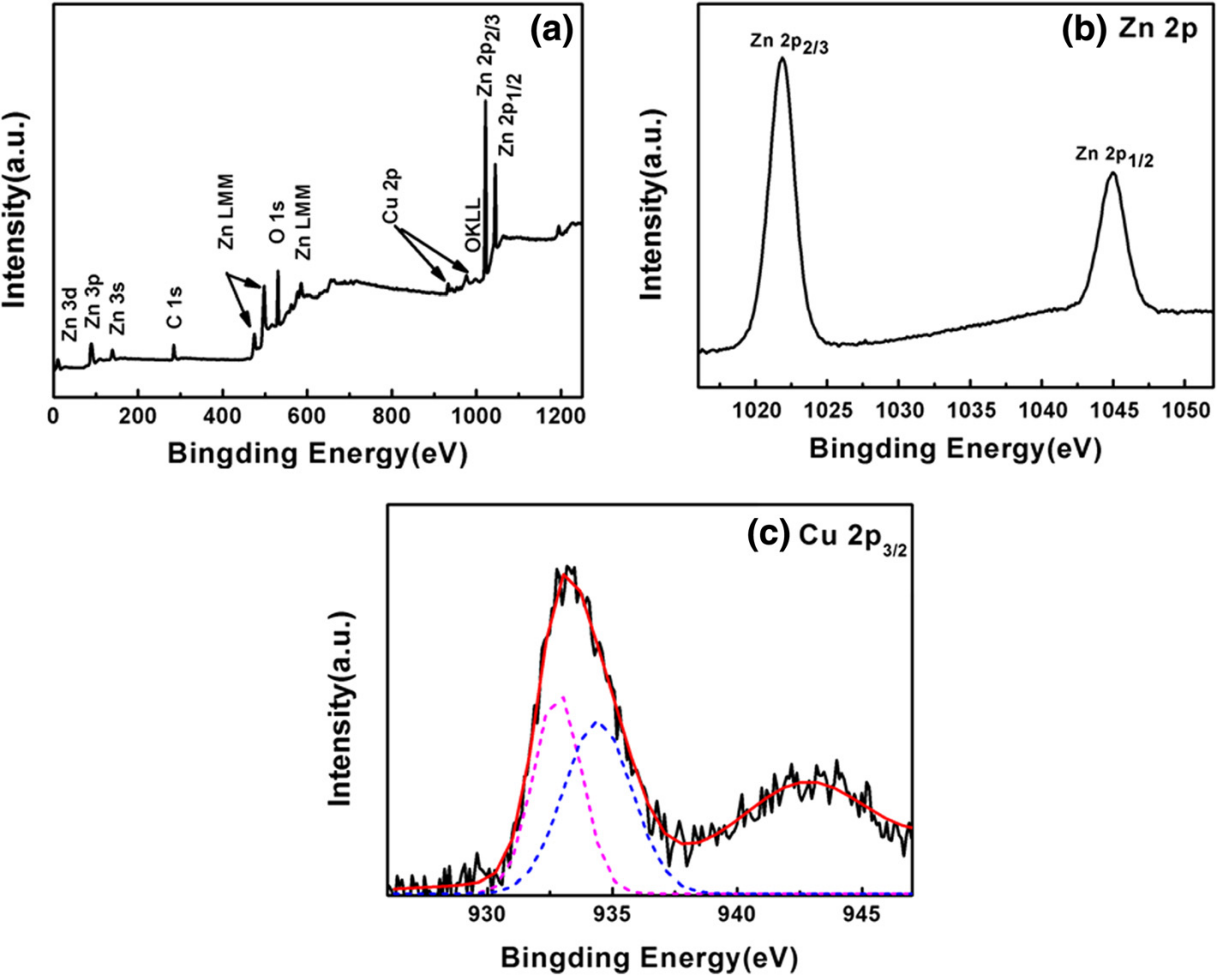
XPS spectra of the PEO-PPO-PEO-capped Cu-ZnO nanoparticles. **(a)** Full XPS spectrum, **(b)** Zn 2p spectrum, and **(c)** Cu 2p_3/2_ spectrum.

The XPS analysis was carried out to analyze the chemical composition of the Cu-ZnO nanoparticle, as shown in Figure 3. The binding energies in the XPS spectra were calibrated using C 1 s peak (284.1 eV). In Figure 3a, all indexed peaks are attributed to C, O, Cu, and Zn, indicating the existence of relevant elements. The presence of carbon arises largely from the surfactant (PEO-PPO-PEO) molecules on the surface of the resulting nanoparticles. Hence, the nanoparticle is mostly composed of three elements, Zn, Cu, and O. Figure 3b shows Zn 2p XPS spectra; the two main peaks observed at the binding energy position of 1,021.8 and 1,044.9 eV correspond to the Zn 2p_3/2_ and Zn 2p_1/2_, respectively, verifying the existence of Zn^2+^. The Cu 2p core-level XPS spectrum of Cu-doped ZnO nanoparticles is shown in Figure 3c. The peaks corresponding to the Cu 2p_3/2_ and 2p_1/2_ core levels were observed at 933.0 and 953.3 eV, respectively, confirming the existence of Cu in the ZnO nanoparticle. The asymmetric peak of Cu 2p_3/2_ can be divided into two peaks by fitting Gaussian: one is the Cu 2p_3/2_ peak of Cu^1+^ centered at 932.80 eV and the other is that of Cu^2+^ centered at 934.41 eV, suggesting that Cu exists in mixed valence state on the surface of this composite material [11,13,15]. Moreover, the atomic ratio of Cu/Zn in the Cu-ZnO nanoparticles has been also calculated from the XPS survey spectra to be about 0.132. The observation is in agreement with the XRD and TEM results as previously addressed.

**Figure 4:**
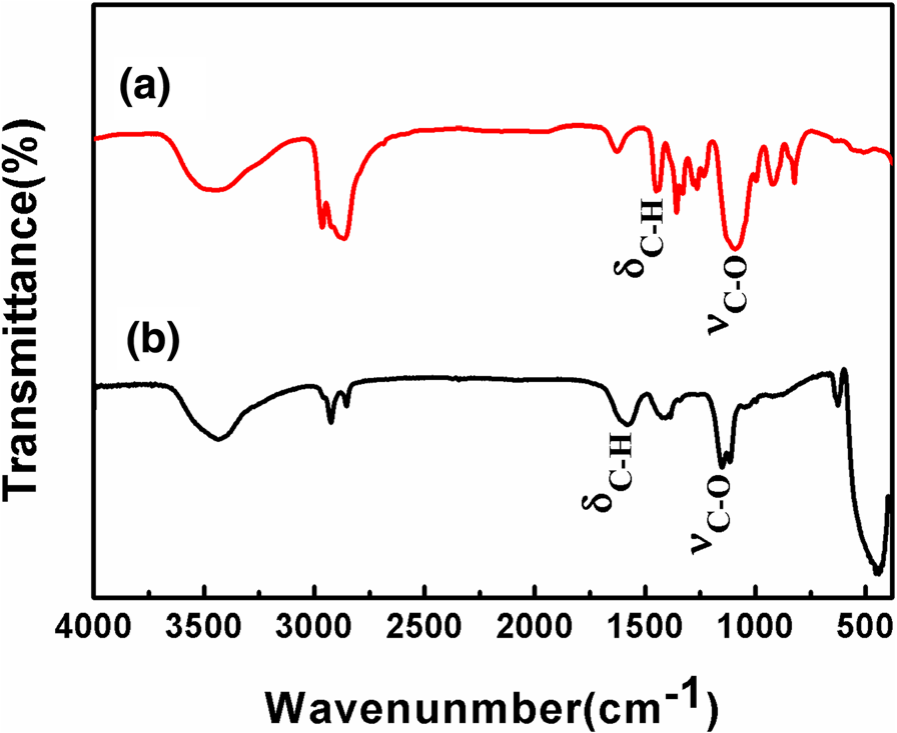
FT-IR spectra of **(a)** the pure PEO-PPO-PEO polymer and **(b)** the PEO-PPO-PEO-capped Cu-ZnO hybrid nanoparticles.

The determination of the existence of PEO-PPO-PEO macromolecules on the surface of the Cu-ZnO hybrid nanoparticles was conducted by FT-IR on the purified nanoparticles in comparison to the pure PEO-PPO-PEO polymer [28,29]. Figure 4 compares the FT-IR spectrum of the purified Cu-ZnO hybrid nanoparticles with that of the pure PEO-PPO-PEO molecules used in the synthesis as the surfactant. In Figure 4a, the pure PEO-PPO-PEO molecules express one strong characteristic band at the position of 1,109.18 cm^−1^ due to the C-O-C stretching vibration of the ether bonding which usually lies in the range of 1,250 to 1,000 cm^−1^ and one sharp characteristic band due to the C-H bending vibration at the position of 1,462.65 cm^−1^ [27,29,34]. As given in Figure 4b, these characteristic vibration and bending features recur in the FT-IR spectrum of the PEO-PPO-PEO-capped Cu-ZnO hybrid nanoparticles, but blue shifting to the positions of 1,160.97 cm^−1^ for the C-O-C stretching vibration and 1,681.87 cm^−1^ for the C-H bending vibration, respectively. Noticeably, the absorption intensities and vibration and bending shapes vary between the pure PEO-PPO-PEO molecules and the PEO-PPO-PEO-capped Cu-ZnO nanoparticles. The shape change and blue shifting in the C-O-C stretching and C-H bending modes may be due to the interactive coordination of the oxygen atoms in the PEO-PPO-PEO main chains to the Cu and Zn atoms in the hybrid nanostructure which could be attributed to changes in the elastic constants of the bonds of the macromolecules sitting on the nanoparticle surface of high curvature owing to the small nanoparticle size and interactions between the macromolecules and the nanoparticle surface [27-29]. Mine while, the double C-H stretching vibrations in the spectra are positioned at 2,883*.*6 and 2,993*.*3 cm^−1^ for the pure PEO-PPO-PEO molecules, but red-shifted to 2,876*.*3 and 2,950*.*7 cm^−1^ for the PEO-PPO-PEO-capped nanoparticles, respectively, which is in striking contrast to the respective blue shifting of the corresponding C-H bending vibration and the C-O-C stretching vibration modes [34]. The observation offers strong evidence that the PEO-PPO-PEO molecules are covered onto the surface of the Cu-ZnO hybrid nanoparticles, as the redundant PEO-PPO-PEO molecules were removed by the purification procedure.

**Figure 5:**
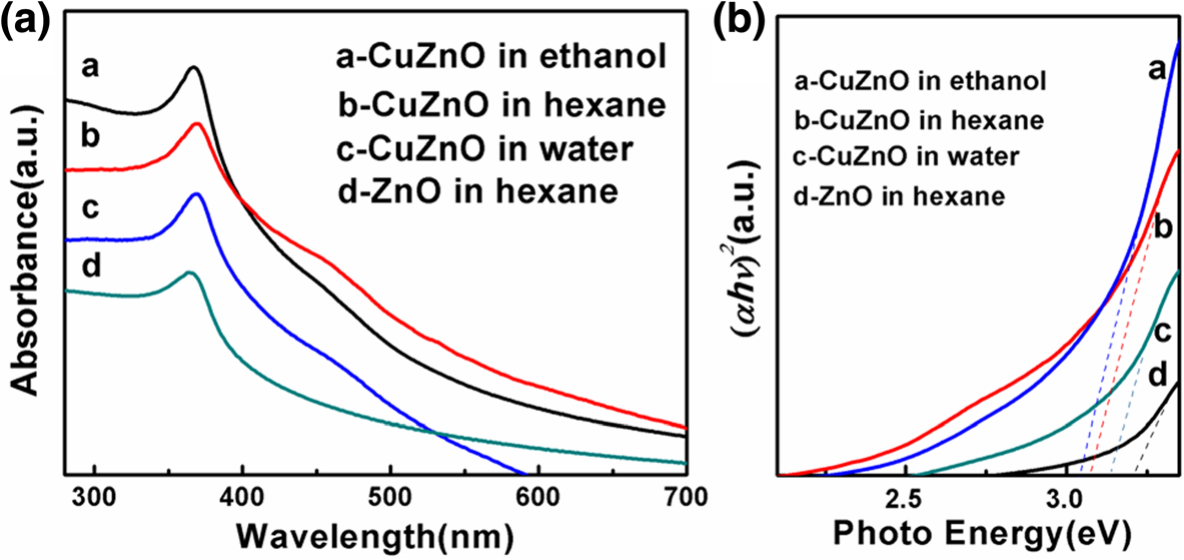
UV–vis spectra and plot of (α*h*ν)^*2*^ vs photon energy for Cu-ZnO hybrid nanoparticles. **(a)** UV–vis spectra of Cu-ZnO hybrid nanoparticles dispersed in different solvents, in comparison to ZnO nanoparticles dispersed in hexane and **(b)** plot of (α*h*ν)^*2*^ vs photon energy for Cu-ZnO hybrid nanoparticles dispersed in different solvents, in comparison to ZnO nanoparticles dispersed in hexane.

The optical properties of the PEO-PPO-PEO-capped Cu-ZnO hybrid nanoparticles were assessed by UV-visible absorption spectroscopy and photoluminescence spectrometry. The PEO-PPO-PEO-capped Cu-ZnO hybrid nanoparticles turn out to be both hydrophilic and hydrophobic, which enable a bi-phase dispersible function intended for an easy transport of the nanoparticles between polar and non-polar solvents without further surface decoration. Figure 5a (a to d) shows the UV–vis spectra of the PEO-PPO-PEO-capped Cu-ZnO hybrid nanoparticles dispersed in ethanol, hexane, and water, together with that of ZnO nanoparticles dispersed in hexane. Apparently, Cu-ZnO hybrid nanoparticles exhibit two kinds of absorption bands. The strong absorption bands observed around 367, 368, and 369 nm for the spectra of the Cu-ZnO hybrid nanoparticles dispersed in ethanol, hexane, and water, respectively, are assigned to the most characteristic absorption of ZnO semiconductor [35], which correspond to red shifting from the absorption band of the ZnO nanoparticles in hexane at the position of ~364 nm. The red shifting of the polymer-capped Cu-ZnO hybrid nanoparticles may be explained in the uniform substitution of Cu ions in the ZnO lattice, the quantum confinement due to the reduced particle dimension, and the solvent effects [29,36,37]. It is worth noting that Cu has a small effect on ZnO absorption due to the close atomic number and similar atomic radius of Cu and Zn. The weak and broad bands around 464, 465, and 468 nm for the spectra of the Cu-ZnO hybrid nanoparticles dispersed in ethanol, hexane, and water, respectively, are attributed to the combinational effect of the narrow band gap attributed to Cu ion (I and II) and wide band gap of ZnO; that is, the absorption bands around 464 to 468 nm of the Cu-ZnO hybrid nanoparticles could be mainly attributed to the strong interaction between the surface oxides of Zn and Cu, while no absorption band of ZnO nanoparticles occurs above 400 nm [9,38,39]. The slight shifting observed in the peak positions in the UV–vis spectra of the polymer-capped Cu-ZnO hybrid nanoparticles dispersed in ethanol, hexane, and water might be caused by the differences in the dielectric constants of solvents. A red shift was observed in the case of aqueous-mediated samples implying bigger particle size of Cu-ZnO. Compared to ethanol and hexane, water molecules have a tendency to generate hydrogen bonds with hydroxyl groups on the Cu-ZnO surface, which hinder the Cu-ZnO dispersion and promote the aggregation of Cu-ZnO nanoparticle, giving rise to bigger particle size [40-42]. The band gap energies of Cu-doped ZnO nanoparticles dispersed in ethanol, hexane, and water, together with that of ZnO nanoparticles dispersed in hexane, were calculated by extrapolating the straight line portion from (α*hv*)^2^ vs. *hv* as shown in Figure 5b (a to d) [43,44]. The changes of the band gap can be seen from Figure 5b clearly. As indicated by Figure 5b, the *E*_g_ value is 3.07, 3.09, and 3.15 eV for Cu-ZnO nanoparticles dispersed in ethanol, hexane, and water, respectively, and 3.22 eV for ZnO nanoparticles dispersed in hexane. All the band gap of the Cu-ZnO nanoparticles were narrowed, revealing that the Cu in the doped ZnO increases the absorption ability of the nanoparticles, and then enhances better photoactivity.

**Figure 6:**
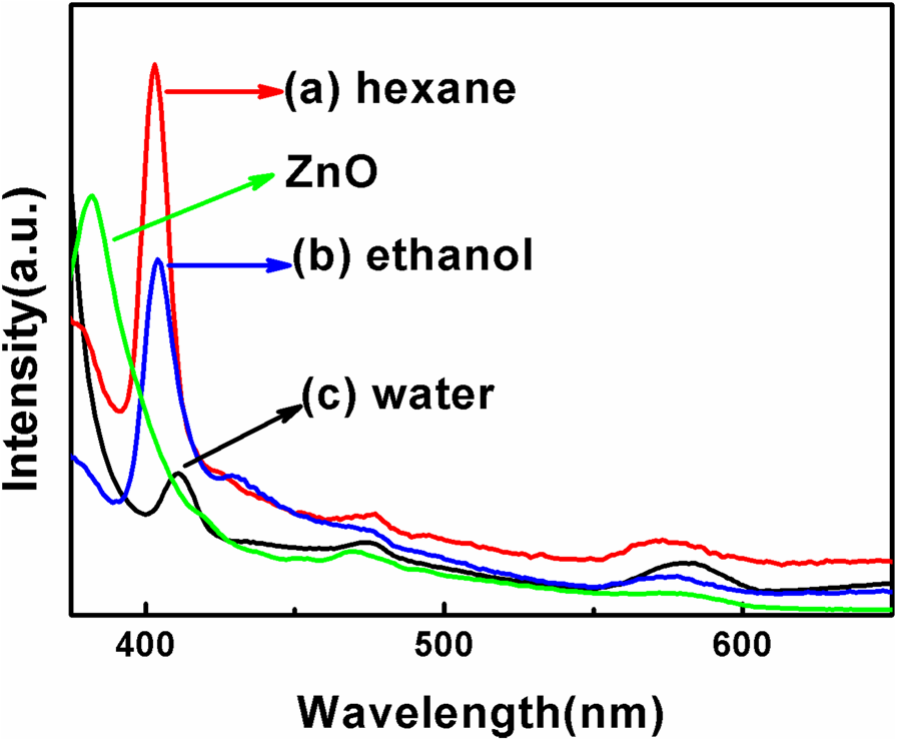
Photoluminescence emission spectra of the Cu-ZnO hybrid nanoparticles dispersed in different solvents. Hexane **(a)**, ethanol **(b)**, and water **(c)**, in comparison to ZnO nanoparticles dispersed in hexane.

Figure 6 shows the PL emission spectra of the PEO-PPO-PEO-capped Cu-ZnO hybrid nanoparticles dispersed in hexane, ethanol, and water, in comparison to that of ZnO nanoparticles dispersed in hexane, examined under the excitation wavelength of 360 nm at room temperature. The UV emission bands around 403, 403, and 410 nm observed for the PEO-PPO-PEO-capped Cu-ZnO hybrid nanoparticles dispersed in hexane (Figure 6a), ethanol (Figure 6b), and water (Figure 6c), respectively, occur from the recombination of the near-band edge (NBE) free exciton [35], indicating a red shift with respect to the emission peak of the ZnO nanoparticles at the position of approximately 381 nm. The red shifting of the Cu-ZnO nanoparticles may be due to the coupling of the band electrons and the localized Cu^2+^ impurity spin [21]. In comparison between the different solvents, the Cu-ZnO nanoparticles dispersed in water show a lower NBE emission compared to those dispersed in hexane and ethanol. This observation could be due to the higher defect concentration in the Cu-ZnO nanoparticles dispersed in water. The weak blue emission bands of Cu-ZnO nanoparticles around 474, 474, and 473 nm in hexane (Figure 6a), ethanol (Figure 6b), and water (Figure 6c), respectively, most likely originate from different deep-level emissions (DLEs), indicating a slightly red shift with respect to that of the ZnO nanoparticles at the position of approximately 471 nm. These DLEs are mainly related to point defects such as the oxygen vacancies and zinc interstitials [45]. The weak green-yellow emission bands of Cu-ZnO nanoparticles around 573 nm in hexane (Figure 6a), 573 nm in ethanol (Figure 6b), and 580 nm in water (Figure 6c), respectively, with respect to that of the ZnO nanoparticles at the position of approximately 573 nm, are typically attributed to the recombination of a delocalized electron close to the conduction band with a deeply trapped hole in the single ionized oxygen vacancy *V*_*o*_^*+*^ and/or the single negatively charged interstitial oxygen ion O_*i*_^*+*^ center [46].

**Figure 7:**
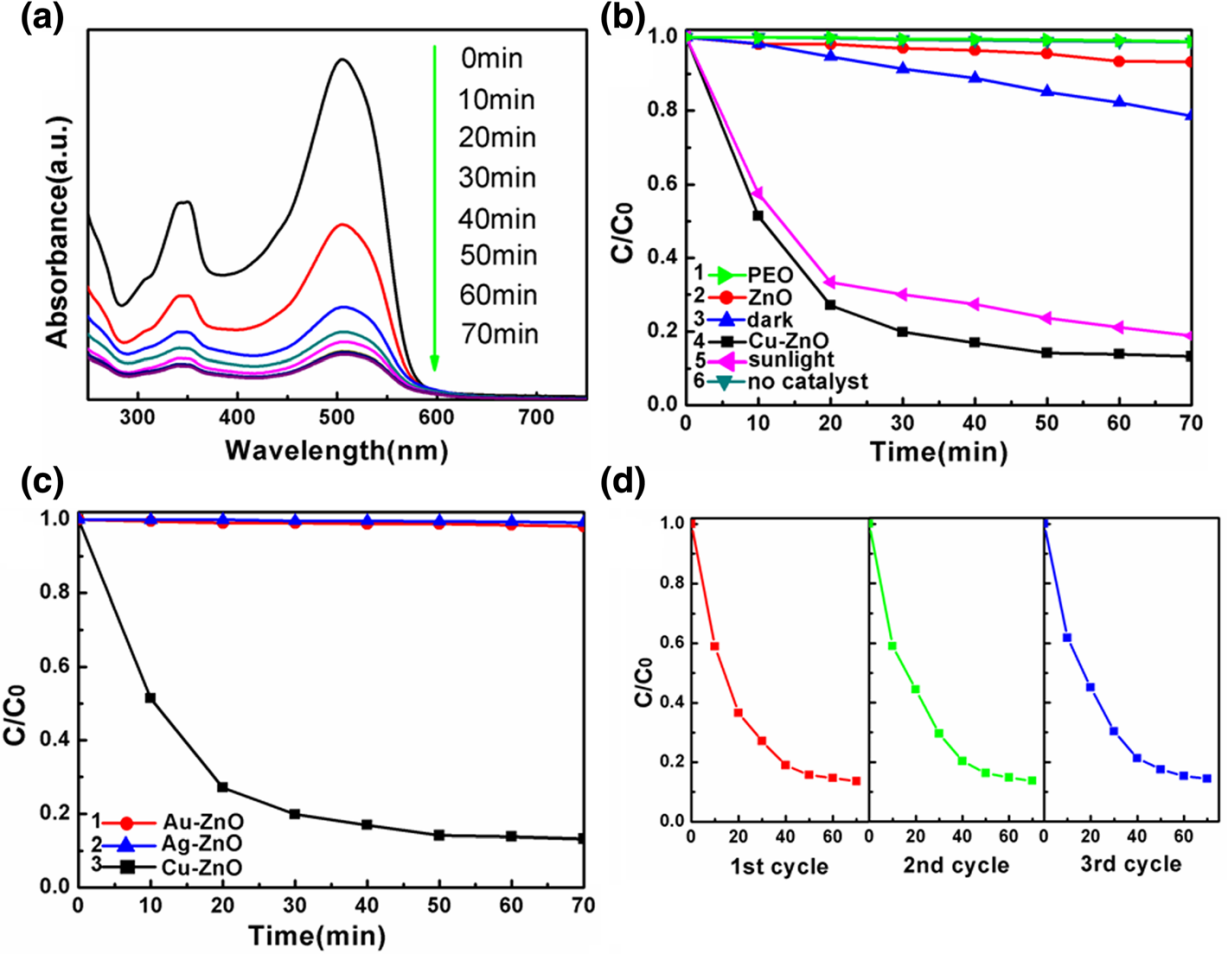
Photo degradation and absorption spectra of Sudan red (III). **(a)** Absorption spectral changes of a Sudan red (III) solution degraded by Cu-ZnO over irradiation time of 0 to 70 min. **(b)** Degradation of Sudan red (III) under different conditions, curves: (1) PEO under UV (2) ZnO under UV, (3) Cu-ZnO in dark, (4) Cu-ZnO under UV, (5) Cu-ZnO under sunlight, (6) no photocatalyst under UV. **(c)** Degradation of Sudan red (III) under different photocatalysts, curves: (1) Au-ZnO under UV, (2) Ag-ZnO under UV, (3) Cu-ZnO under UV. **(d)** Reusability of Cu-ZnO catalyst for the degradation of Sudan red (III) for three cycles.

Sudan dyes (Sudan I, II, III, IV, and Para Red) are azo compounds commonly used to confer color to various materials such as oils, solvents, gasoline, and inks [47]. Due to their low price and intense red color, these compounds are also used as food dye, mainly in paprika powders, curry, and chili, as well as palm oil, in order to intensify the color. However, azo dyes have demonstrated significant carcinogenic and mutagenic properties and, therefore, are categorized as class 3 carcinogens by the International Agency for Research on Cancer (IARC). Nevertheless, Sudan dyes have been found recently in numerous food products and cosmetics [47,48]. Thus, the elimination or reduction of these dye pollutants is imperative for toxicity, mutagenicity, and carcinogenicity of the azo dyes. The degradation of Sudan red (III) can be conveniently monitored by optical absorption spectroscopy. Figure 7a shows the absorption spectra of the Sudan red (III) solution containing the Cu-ZnO hybrid nanoparticles at different intervals after exposing the solution under UV–vis irradiation, in which the intensity of the characteristic absorption of Sudan red (III) at 513 nm decreases with the increasing of the exposure time. The reduced intensity in the main absorption peaks is due to the degradation of Sudan red (III). In Figure 7b, the photo degradation of Sudan red (III) was investigated in the presence of Cu-ZnO under UV irradiation and sunlight and in dark, compared with ZnO, PEO, and no photocatalyst under UV irradiation. The *y*-axis is referred as *C*/*C*_0_ in which *C* is the concentration of Sudan red (III) at each irradiated time interval determined at *λ*_max_ while *C*_0_ is the starting concentration when adsorption-desorption equilibrium was achieved. As shown in Figure 7b, Sudan red (III) decomposes 86.8% (curve 4), 81.2% (curve 5), and 21.5% (curve 3), respectively, when Cu-ZnO acts as the photocatalyst under UV irradiation and sunlight and in dark after 70 min, while only 6.7% (curve 2), 0.12% (curve 1), and 0.13% (curve 6) degradation are observed in the presence of ZnO under UV irradiation, PEO under UV irradiation, and barely exposed only with UV light (photolysis). PEO-PPO-PEO capping on the surface of Cu-ZnO enables well dispersibility of the Cu-ZnO nanoparticles both in non-polar and polar solvent, though it shows no influence in the adsorption and degradation of Sudan red. The result that the photocatalytic performance of Cu-ZnO in dark is better than no catalyst revealed that the photocatalytic reactions were induced by the catalyst. The Cu-doping resulted in a significant enhancement in the photocatalytic activity compared with pure ZnO both under UV irradiation and sunlight. More interesting, Cu-ZnO even exhibit better photocatalytic performance in dark than ZnO under UV irradiation.

The mechanism of photoinduced molecular transformation or reaction in ZnO photocatalytic system is based on the generation of electron–hole pairs. When light with energy higher or equal to the band gap energy is irradiated to the ZnO surface, the transition of photoelectrons from the valence band (VB) to the conduction band (CB) may be promoted, leaving behind a hole in the valence band. The photoelectrons may be captured by the O_2_ on the surface of ZnO for the formation of O_2_^•−^ radicals, while the holes react with ethanol in order to generate ethoxy radicals. The ethoxy radicals and O_2_^•−^ radicals are found to be major species responsible for organic dye degradation [49,50]. The weak photocatalytic activity of ZnO nanoparticles may be due to the quick recombination of photo-generated charge carries. The Cu-ZnO hybrid nanoparticles likely result in a lower band gap, which is conducive to enhanced photocatalytic activity. At the same time, the Cu^2+^ ions with half-filled electronic configuration could capture the photogenerated electrons and reinforce the separation efficiency of photogenerated electron–hole pairs in ZnO, which contribute to the improvement of the photocatalytic activity of Cu-ZnO nanoparticles [51-53]. The Cu-ZnO shows better photocatalytic efficiency even in dark may be due to the oxidation of Cu ions on the surface of nanoparticle catalyst [54-56].

Recycling experiments of the Cu-ZnO catalyst was carried out under UV irradiation. The Cu-ZnO catalyst was regained from the degradation mixture by filtration and was then washed with ethanol. The recovered catalyst was reused for the photodegradation of Sudan red (III) under the identical reaction conditions. The catalytic activity of Cu-ZnO was tested for three times, as shown in Figure 7d, revealing that even after being reused for three times, the Cu-ZnO exhibited photocatalytic behavior and the Sudan red (III) dye degradation efficiency was practically the same. The recyclability of Cu-ZnO is attributed to the resistance and stability to photocorrosion, which is desired for a greener and environment-friendly approach.

We compared the photocatalytic behavior of Cu-ZnO nanoparticles with Au-ZnO and Ag-ZnO nanoparticles which both exhibit enhanced photocatalyst activity. [28,29]. As shown in Figure 7c, it is clear that the catalyzing activity of Cu-ZnO is much stronger than that of Au-ZnO and Ag-ZnO under the same conditions (UV irradiation). Consequently, Cu-ZnO was found to be a better photocatalyst than ZnO, Au-ZnO, and Ag-ZnO and capable of working under sun light/visible light irradiation. Thus, the Cu-ZnO nanoparticles are favorable candidates for potential application as a promising photocatalyst.

## Conclusions

In summary, we have synthesized the Cu-ZnO hybrid nanoparticles by one-pot non-aqueous nanoemulsion process in the presence of PEO-PPO-PEO polymer. The FT-IR assessment confirms that the hydrosoluble, non-toxic, and stable PEO-PPO-PEO macromolecules are present on the surface of the nanopareicles. The morphological and structural analyses demonstrate the narrow particle size distribution with an average size of approximately 19.4 nm in diameter and high crystallinity of Cu-ZnO hybrid nanoparticles. The XPS confirms Cu^1+^ and Cu^2+^ in ZnO. The optical measurements show well-defined absorption bands and emission bands for the nanoparticles dispersed in organic and aqueous solvents. The photocatalytic activity for Sudan red (III) dye degradation reveals that the Cu-ZnO catalyst exhibits higher photocatalytic efficiency than ZnO, and its photocatalytic efficiency is practically the same after three cycles of use. Such bi-phase dispersible Cu-ZnO hybrid nanoparticles could be applicable in photocatalysis.
